# Asthma diagnosis and treatment – 1028. A rare case of bronchial asthma and allergic rhinitis with complete right lung agenesis investigated in tertiary case hospital

**DOI:** 10.1186/1939-4551-6-S1-P27

**Published:** 2013-04-23

**Authors:** Ram Awadh Singh Kushwaha, Rajiv Garg, TG Rangnath, Rajendra Prasad, Surya Kant

**Affiliations:** 1 Pulmonary Medicine, K G Medical University up Lucknow, Lucknow, India; 2 Up RIMS & R, Saifai, Etawah, SAIFAI, ETAWAH, India

## Background

Unilateral lung agenesis is extremely rare. These patients usually present in infancy or childhood with recurrent respiratory infection and cardiopulmonary insufficiency. Here, we report a rare case of bronchial asthma and allergic rhinitis whose investigation showed complete right lung agenesis.

## Methods

A 26 year-old lady presented with episodic breathlessness, chest tightness, recurrent nasal obstruction and excessive sneezing, mainly during change of season along with opacity of the right hemithorax on chest x-ray. Further detailed work-up including spirometry, high resolution CT scan of the thorax and fibreoptic bronchoscopy confirmed complete right lung agenesis in patients with bronchial asthma and allergic rhinitis.

## Results

Chest x-ray PA view showed homogeneous opacity with signs of volume loss on the right side. After that we confirmed by CT images of the thorax that revealed complete absence of right lung with hyperinflation and herniation of the left lung to the right side. Further, fiberoptic bronchoscopy showed completely absent right main bronchus and trachea directly leading to left main bronchus. Spirometry was also performed which revealed obstructive airway disorder with 13% post bronchodilator reversibility confirming the diagnosis of bronchial asthma. Complete control of symptoms was achieved with formoterol 6 μg and mometasone 200 μg (via dry powder inhaler) and intranasal fluticasone 50 μg (nasal spray) 2 puffs twice daily and oral montelukast 10 mg with levocetirizine 5 mg once daily. The patient was completely asymptomatic when reviewed after 1 month follow up and she was advised to stop intranasal fluticasone and to continue formeterol and mometasone inhaler with oral montelukast and levocetirizine preparation.

## Conclusions

This rare case illustrates that a high index of suspicion is necessary to diagnose the condition. Simple and regular asthma medications may good enough to relieve symptoms even in patients with unilateral lung agenesis for control of symptoms.

